# Cyclotide host-defense tailored for species and environments in violets from the Canary Islands

**DOI:** 10.1038/s41598-021-91555-y

**Published:** 2021-06-14

**Authors:** Blazej Slazak, Klara Kaltenböck, Karin Steffen, Martyna Rogala, Priscila Rodríguez-Rodríguez, Anna Nilsson, Reza Shariatgorji, Per E. Andrén, Ulf Göransson

**Affiliations:** 1 W. Szafer Institute of Botany, Polish Academy of Science, 46 Lubicz St., 31-512 Kraków, Poland; 2 Department of Pharmaceutical Biosciences, Pharmacognosy, Uppsala University, Biomedical Centre (BMC) 574, 75123 Uppsala, Sweden; 3 Medical and Pharmaceutical Biotechnology, IMC University of Applied Sciences, 3500 Krems, Austria; 4 Department of Plant Physiology and Biochemistry, Faculty of Biochemistry, Biophysics and Biotechnology, Jagiellonian University, Gronostajowa 7, 30-387 Kraków, Poland; 5 Instituto Universitario de Estudios Ambientales y Recursos Naturales (IUNAT), Universidad de Las Palmas de Gran Canaria, Campus Universitario de Tafira, 35017 Las Palmas de Gran Canaria, Canary Islands Spain; 6 Department of Pharmaceutical Biosciences, Medical Mass Spectrometry Imaging, Uppsala University, Biomedical Centre (BMC) 591, 75124 Uppsala, Sweden; 7 Science for Life Laboratory, Spatial Mass Spectrometry, Uppsala University, Biomedical Centre (BMC) 591, 75124 Uppsala, Sweden

**Keywords:** Biochemistry, Chemical biology, Ecology, Evolution, Plant sciences

## Abstract

Cyclotides are cyclic peptides produced by plants. Due to their insecticidal properties, they are thought to be involved in host defense. Violets produce complex mixtures of cyclotides, that are characteristic for each species and variable in different environments. Herein, we utilized mass spectrometry (LC–MS, MALDI-MS), transcriptomics and biological assays to investigate the diversity, differences in cyclotide expression based on species and different environment, and antimicrobial activity of cyclotides found in violets from the Canary Islands. A wide range of different habitats can be found on these islands, from subtropical forests to dry volcano peaks at high altitudes. The islands are inhabited by the endemic *Viola palmensis*,* V. cheiranthifolia*,* V. anagae* and the common *V. odorata.* The number of cyclotides produced by a given species varied in plants from different environments. The highest diversity was noted in *V. anagae* which resides in subtropical forest and the lowest in *V. cheiranthifolia* from the Teide volcano. Transcriptome sequencing and LC–MS were used to identify 23 cyclotide sequences from *V. anagae*. Cyclotide extracts exhibited antifungal activities with the lowest minimal inhibitory concentrations noted for *V. anagae* (15.62 μg/ml against *Fusarium culmorum*). The analysis of the relative abundance of 30 selected cyclotides revealed patterns characteristic to both species and populations, which can be the result of genetic variability or environmental conditions in different habitats. The current study exemplifies how plants tailor their host defense peptides for various habitats, and the usefulness of cyclotides as markers for chemosystematics.

## Introduction

The term cyclotide is a combination of the words *cyclic peptide* and was introduced in 1999^1^. At that time, only around 30 of these plant peptides had been discovered, including the prototypic kalata B1 from *Oldenlandia affinis* Rubiaceae^2^. However, already then, the largest number of cyclotides had been isolated from Violacae^3–5^. To date, hundreds of different cyclotides have been described from six angiosperm families: the Rubiaceae, Cucurbitaceae, Fabaceae, Solanaceae, Poaceae and Violaceae^2,5–10^. These peptides are around 30 amino acids long and display the signature cyclic cystine knot motif: a cyclic backbone containing six cysteine residues forming three disulfide bonds in conserved positions^11^. Many cyclotides are unique to certain plant species, but some are expressed by more than one species^8,12^. Therefore, it is hypothesized that they evolved independently in non-related plant families^13^.

Cyclotides can prove to be valuable as chemotaxonomic markers in the plant families that express them. All the species from Violaceae family surveyed in Burman et al.^8^ appeared to produce cyclotides. Moreover, the study found that each species has a unique suite of these peptides, not found in the others. The study indicated that species can be distinguished from one another by their cyclotide composition^8^. Another study supports this hypothesis, showing the distinct cyclotide production patterns among species of Australian species from genus *Hybanthus* (Violeaceae)^14^.

Plants accumulate cyclotides in high quantities and this requires many resources^2,15^. Their abundance implies that they play key biological roles. Cyclotides are cell membrane disruptive peptides and have been shown to be active against insect larvae, nematodes, bacteria, fungi, and both plant and mammalian cells^16–22^. Based on their wide range of bioactivities, it was suggested that cyclotides are involved in plant host defense^22^. Cyclotides with different amino-acid sequences show differences in specific biological properties. Indeed, it has been suggested that plants produce peptides targeting specific pathogens or pests^23^.

It has not been established yet whether cyclotides are part of constitutive defense, or if their production is regulated by stress or environmental factors. The study by Mylne et al.^24^ showed that the expression levels of five cyclotide genes in *Oldenlandia affinis* were not altered by in response to various kinds of stress. This suggested that cyclotides in *O. affinis* are part of an innate defense. However, a number of studies indicate that the expression patterns in various plants change with season, in different habitats and also in response to plant growth regulators and biological elicitors including chitosan^14,15,25,26^. It has been suggested that some plants express a “basic set” of cyclotides continuously, whereas production of additional, more specific ones is switched on as needed^26^.

The Canary Islands volcanic archipelago is situated in the Atlantic Ocean, 100 km off the Moroccan coast (northwest Africa). Due to isolation among islands and topographic and environmental characteristics, the archipelago has a rich flora with around ~ 610 species^27^. The islands comprise a high variety of climatic and environmental conditions, from subtropical laurel forests to high altitude, dry and cold volcanic peaks. Therefore, the Canaries are considered to be within the Mediterranean Basin biodiversity hotspot^28^. Because of this, the Canary Islands are an ideal place not only to look for novel cyclotides but also to study how their production patterns vary between species and populations inhabiting different environments.

Seven species from the genus *Viola* (Violaceae) have been described in the Canary Islands*: Viola odorata*,* V. riviniana V*,* arvensis* and *V. kitaibeliana*, considered as native, and *Viola palmensis*,* V. cheiranthifolia*,* V. anagae* that are endemic to the islands^27^. Recently, the new species *V. guaxarensis* has been described, due to morphological and genetic differences from *V. cheiranthifolia*^29,30^, previously considered as a population of the later (the “Guajara” population)^27^.

In the present article, we focus on the three endemic species (*V. palmensis*, *V. cheiranthifolia* and *V. anagae*) and the commonly occurring *V. odorata*. None of the three violets endemic to the Canary Islands have previously been examined for their cyclotide expression. *V. odorata* is a model plant in cyclotide research. This is because its peptides and their biological activities, roles and distribution across tissues and organs have been the subject of more than a dozen studies^1,20,31–33^. These four species belong to two different subsections of the genus *Viola*: *V. anagae* and *V. odorata* to the section *Viola*, and *V. cheiranthifolia* and *V. palmensis* to section *Melanium*^30,34–36^. Both sections are well defined phylogenetically and morphologically^36,37^, being commonly recognized as violets (sect. *Viola*) and pansies (sect. *Melanium*). They derived from allopolyploidization events from two major clades that diverged around 20 million years ago^36^. *V. palmensis* and *V. cheiranthifolia* are found on volcanic rock at altitudes above 1800 m a.s.l on La Palma and 2300 m a.s.l. in Tenerife respectively, whereas *V. anagae* grows in subtropical laurel forest of the Anaga Rural Park, Tenerife^30,35,38^. These three species have restricted distributions, and are classified as vulnerable on the Red List of Spanish Vascular Flora^39^.

In the current study, we investigate the cyclotides produced in violets from the Canary Islands, their diversity, activity against fungal pathogens and variability between species and populations. We evaluate how the peptide defenses are shaped for different environments and the applicability of cyclotides as chemosystematic markers.

## Results

### Cyclotide diversity in violets from the Canary Islands

The diversity of cyclotides produced by four violets (*V. odorata*, *V. anagae*, *V. palmensis* and *V. cheiranthifolia*) was explored using MALDI-MS. The highest number of different peptides were found in *V. anagae* and *V. odorata*—41 and 37, respectively. Of these, more than 60% were unique to the single species and not found in the other violets tested, judged by their monoisotopic molecular mass (Table 1). A substantially lower number of cyclotides could be identified in *V. palmensis* (25 individual cyclotides) and *V. cheiranthifolia* (26) with eight and six masses observed exclusively in those species, respectively. *V. palmensis* and *V. cheiranthifolia* had fifteen peptides in common and only 11 masses were found in both *V. anagae* and *V. odorata* (Table 1). All monoisotopic masses of cyclotides found in each species are shown in Supplement 1.

**Table 1:** Total number and number of unique monoisotopic molecular masses (individual cyclotides) found in the mass spectra of extracts from different violets.

Species	Number of cyclotides	Unique to species
*V. anagae*	41	25
*V. odorata*	37	27
*V. palmensis*	25	8
*V. cheiranthifolia*	26	6

### Cyclotide sequences in *V. anagae*—de novo MS/MS sequencing and transcriptome analysis

**Figure 1:**
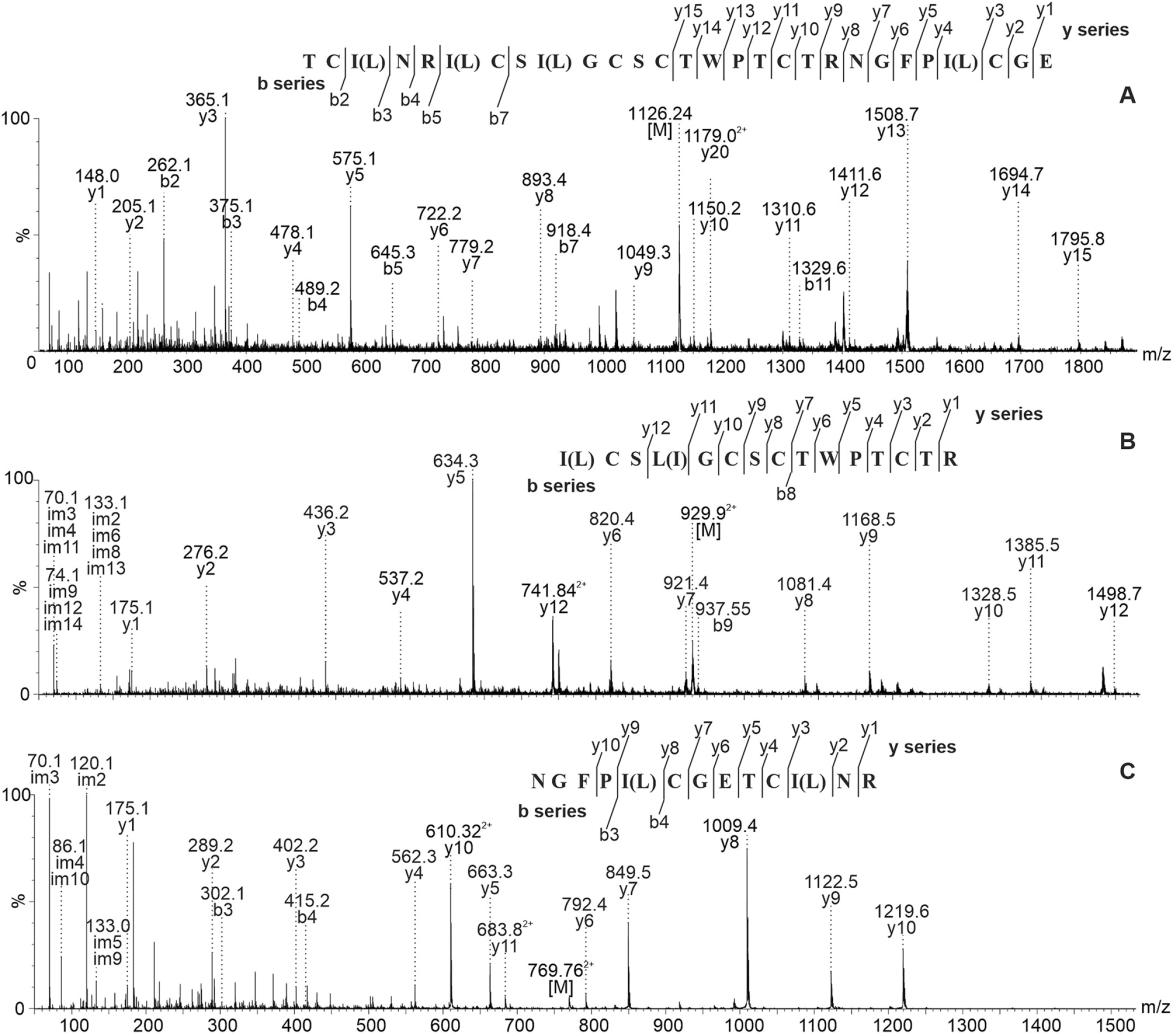
De novo MSMS sequencing of a peptide with observed monoisotopic molecular mass of 3010.24 isolated from *V. anagae*—fragmentation patterns of peptides resulting from GluC (**A**) and trypsin (**B**,**C**) cleavage.

A cyclotide with monoisotopic molecular mass 3010.24 was isolated from *V. anagae*, enabling MS/MS de novo sequencing. Cleavage with endoproteinase GluC resulted in a single linear fragment with [M+3H]^3+^ 1126.57, 15 y-ion and five b-ions could be assigned in the MS/MS fragmentation pattern (Fig. 1A). Tryptic cleavage resulted in two fragments with [M+2H]^2+^ 929.9 and [M+2H]^2+^ 769.87, the MS/MS fragmentation of those allowed to identify the rest of the peptide’s amino acid sequence (Fig. 1B,C). The peptide was named vian 2 and its complete sequence is shown in Table 2. Database searches in the CyBase cyclotide database (http://www.cybase.org.au) and protein BLAST in Uniprot database verified the novelty of the sequence.

**Table 2:**
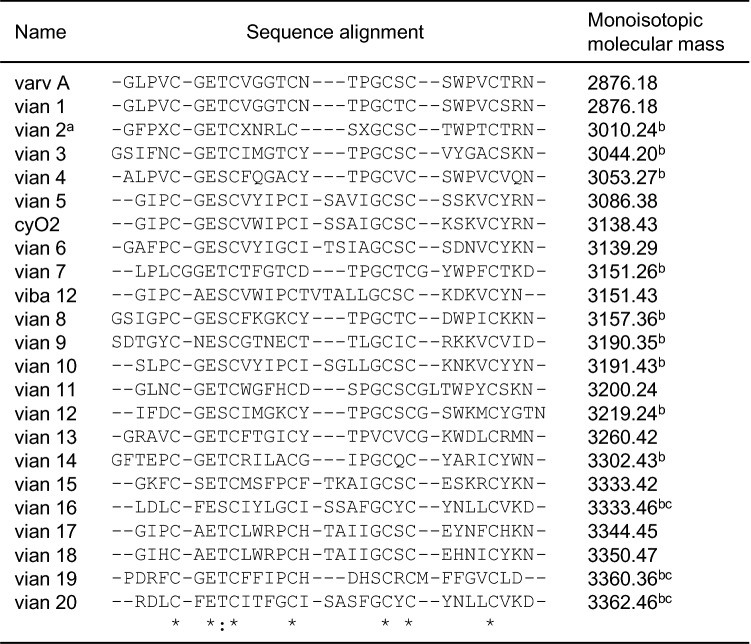
Sequence alignment of the novel, not described previously (vian 1–20) and known (varv A, cyO2, viba 12) cyclotides found in *V. anagae*.

Novelty or identity of the sequences has been established by comparing them to sequences deposited in Cybase (http://www.cybase.org.au) and Uniprot (https://www.uniprot.org/).

^a^Sequenced by MSMS on the peptide level. X in the sequence stands for either I or L.

^b^Masses not found in mass spectra—undetermined N-terminal cleavage position from the precursor peptide.

^c^The peptide tail sequence recognized by the cyclization enzyme is present in the precursor protein.

*V. anagae* transcriptome analysis yielded 22 cyclotide sequences of which 19 were new and three were known (Table 2). The new peptides were named using an established nomenclature^40^. Additionally, 22 precursor peptides were characterized; the nucleotide sequences of the transcripts together with translated open reading frames containing cyclotides are shown in Supplement 2.

### Cyclotide extracts display antifungal activity

**Figure 2:**
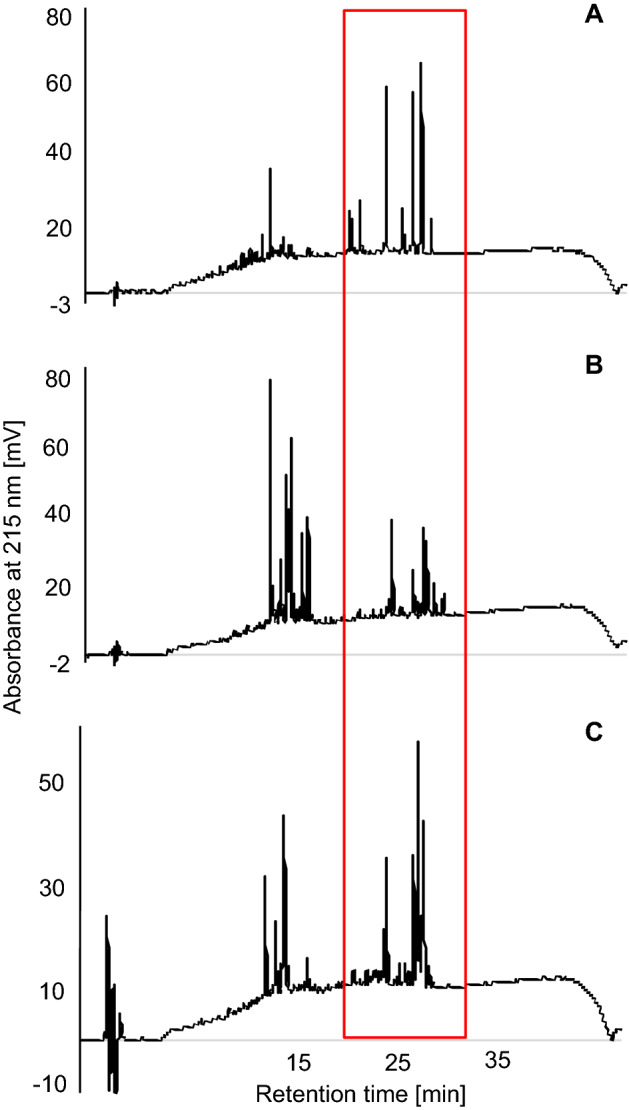
Chromatograms of the cyclotide extracts used in the antifungal assays: *V. anagae* (**A**), *V. cheiranthifolia* (**B**), *V. palmensis* (**C**). The region of the chromatogram containing cyclotides was marked with a frame.

*V. anagae* and *V. palmensis* appeared to contain more cyclotides and fewer other compounds compared to *V. cheiranthifolia.* Higher peaks in the region of the chromatogram where the peptides were expected were observed in HPLC trace (Fig. 2). An extract of *V. anagae* showed the highest activity against three out of the five tested fungal species: *M. fragariae*,* B. cinerea*,* F. culmorum* (Table 3). The cyclotide extract was most effective against *F. culmorum* with MIC of 15.62 µg ml^−1^. The activity was weaker by one (MIC: 31.25 µg ml^−1^) and four dilution factors (MIC: 125 µg ml^−1^) respectively against *B. cinerea* and *M. fragariae*. None of the tested extracts were active against the *Aspergillus* strains (Table 3). *V. palmensis* extract showed some activity against *F. culmorum* (500 µg ml^−1^), whereas the extract of *V. cheiranthifolia* was not active against any strain at the tested concentrations.

**Table 3:** The minimal inhibitory concentration (MIC) of three Violaceae species’ cyclotide extracts against five fungal plant pathogens.

	MIC (µg/ml)		
	*V. anagae*	*V. palmensis*	*V. cheiranthifolia*
*A. fumigatus*	> 400	> 400	> 1000
*A. pseudonomius*	> 400	> 400	> 1000
*M. fragariae*	125	> 500	> 1000
*B. cinerea*	31.25	> 500	> 1000
*F. culmorum*	15.62	500	> 1000

### Differences in cyclotide expression between species and locations

**Figure 3:**
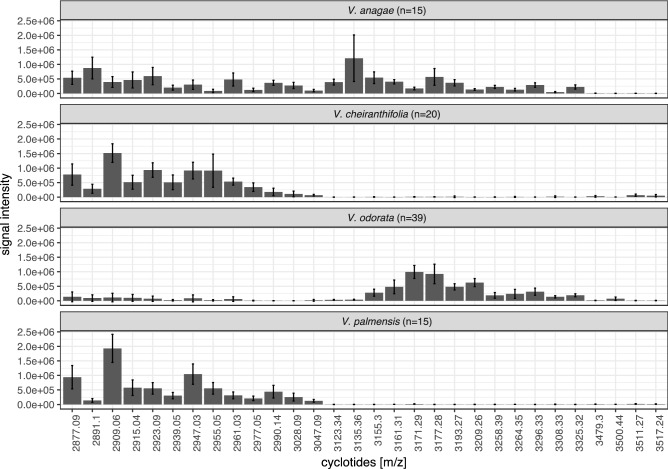
Bar-plot analysis of the mean relative abundances (ion intensity per pixel per spot) of 30 selected cyclotides, across all the investigated violet species: *V. anagae*,* V. cheiranthifolia*,* V. odorata*,* V. palmensis.* Bars indicate SD. *n* number of individual plants.

MALDI-MS analysis of extracts showed peptide patterns characteristic for particular species as illustrated in Fig. 3. The profiles of *V. cheiranthifolia* and *V. palmensis* show similarities, which is also notable in the LC-UV traces in Fig. 2. In contrast, *V. anagae* and *V. odorata* exhibited more dissimilar cyclotide patterns (Fig. 3).

**Figure 4:**
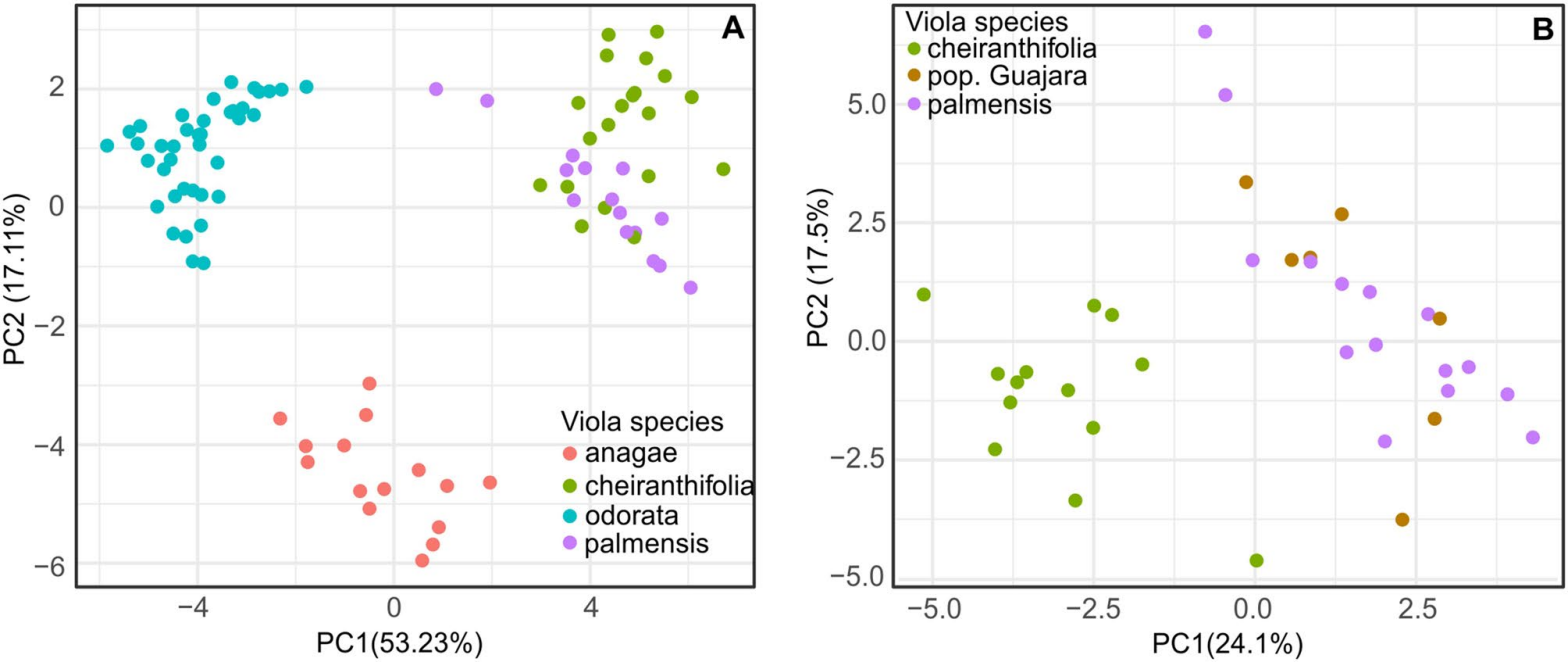
PCA analysis of the relative abundances of 30 selected cyclotides, across all the investigated violet species: *V. anagae*,* V. cheiranthifolia*,* V. odorata*,* V. palmensis* (**A**); and violet species from section Melanium: *V. cheiranthifolia* (including population Guajara) and *V. palmensis* (**B**). Single point indicates individual sampled plant.

The principal component analysis (PCA) was based on the relative abundance of 30 selected cyclotide masses. As shown in Fig. 4A, samples grouped by species in the first two components account for the majority of variation in the data (~ 70%). In addition, species belonging to different sections of genus Viola (*Viola* or *Melanium*) formed separate groups. *V. odorata* and *V. anagae* (section *Viola*) were clearly differentiated from the other two tested species along the first component (PC1), whereas *V. anagae* differed from the other samples along the PC2 axis. The species from section *Melanium*,* V. cheiranthifolia* and *V. palmensis*, showed small differences in their cyclotide production patterns and occupy the same space in the PCA (Fig. 4A). However, the separation of these species became apparent in PCA analysis including only different populations of *V. cheiranthifolia* and *V. palmensis* (Fig. 4B). One exception was noted from this trend—the Guajara population, isolated from other populations in PCA space, and clustered with *V. palmensis* (Fig. 4B).

**Figure 5:**
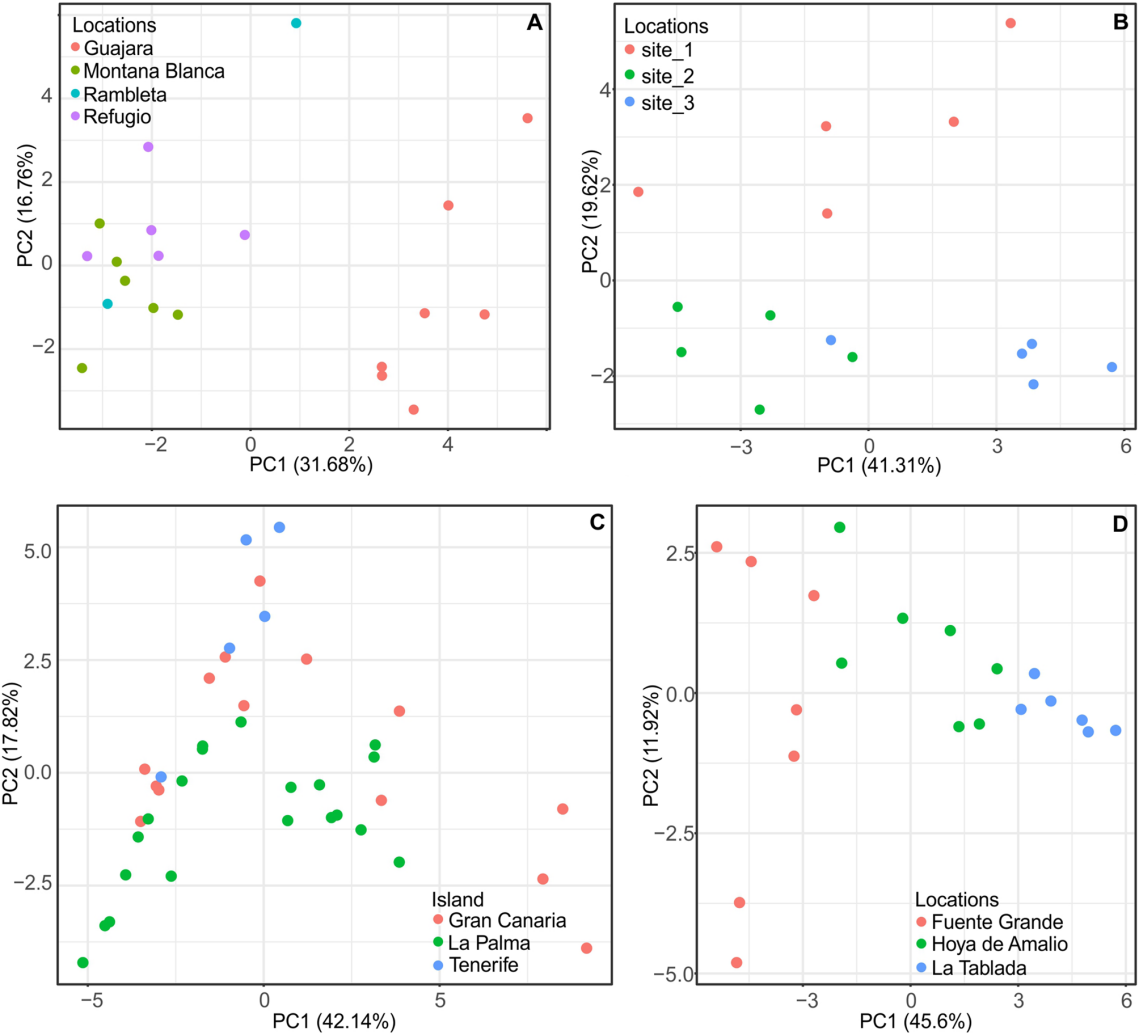
PCA analysis of the average relative abundances of 30 selected cyclotides across different populations of *V. cheiranthifolia* (**A**), *V. anagae* (**B**); and different populations of *V. odorata:* by island of origin (**C**) and for populations from La Palma (**D**). Single point indicates individual sampled plant.

**Figure 6:**
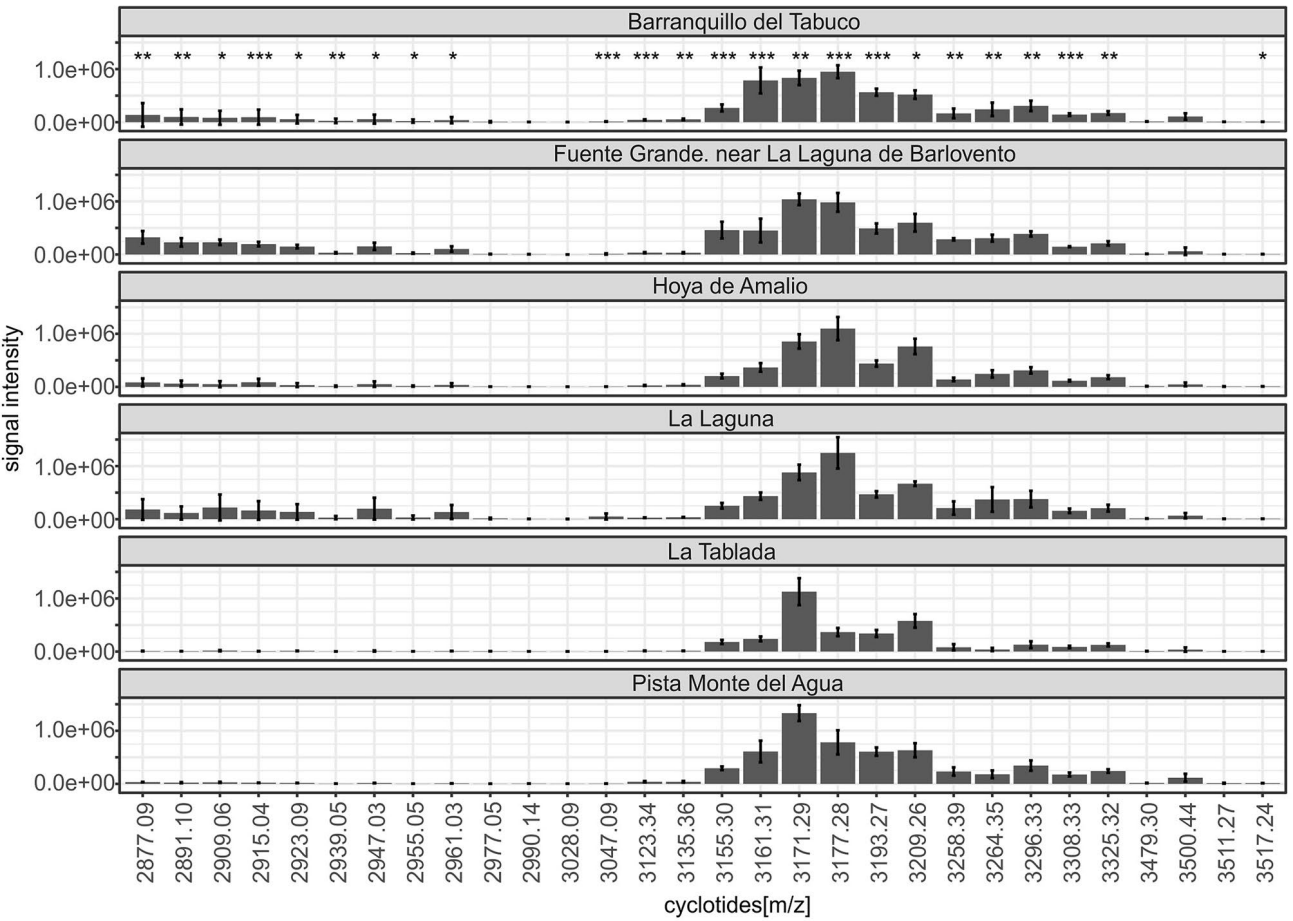
Average relative abundances of the 30 selected cyclotides, across different populations of *V. odorata* (Barranquillo del Tabuco n = 7, Fuente Grande n = 7, Hoya de Amalio n = 7, La Laguna n = 7, La Tablada n = 6, Pista Monte del Agua n = 5). Statistical significance (Kruskal–Wallis tests) of differences in cyclotide relative abundances among any of the populations/sample localities is denoted by asterisks (p < 0.05*, p < 0.01**, p < 0.001***).

The PCA for *V. cheiranthifolia* plants from different sites confirmed the separation of the Guajara population in terms of the cyclotide production patterns (Fig. 5A). PCA prepared for *V. palmensis* populations did not show any significant differences between them. Minor levels of variability, resulting in separation of specific populations in PCA, was observed in case of *V. anagae* and *V. odorata* (Fig. 5B–D). Additionally, PCA for *V. odorata* showed some degree of variation between populations from the three sampled islands and among the ones from La Palma (Fig. 5C,D). Statistical analyses of differences between populations showed significance (*p *values < 0.05) in 15 of the 30 analyzed cyclotides for *V. anagae* and *V. cheiranthifolia*. The differences between *V. odorata* populations were significant for 24 out of 30 analyzed cyclotides (Fig. 6). Additional PCA analyses are presented in Supplement 3.

## Discussion

The current work revealed a variety of novel cyclotides in violets endemic to the Canary Islands, *V. anagae*, *V. cheiranthifolia* and *V. palmensis.* None of these species have been previously investigated for their peptide constituents. These three endemic violets are rare and inhabit small defined areas, and both the number of populations and individual plants are limited^30,35^. All species were analyzed by MALDI-MS, antifungal assays, and HPLC fingerprinting, but only *V. anagae* was chosen for MSMS de novo peptide sequencing and transcriptome sequencing due to better availability of plant material from a larger population accessible all year, independent of season. It was also the violet from which we expected the highest number of different cyclotides based on preliminary analyses of mass spectra.

The peptide vian 2 (monoisotopic molecular mass of 3009.06 Da) was isolated and sequenced with MSMS. This peptide has a proline in loop five and therefore belongs to the Möbius subfamily^1^. When compared to known cyclotide sequences deposited in Cybase (http://www.cybase.org.au/; Mulvenna et al*.*^41^), the peptide loops one, three and four were similar in length and sequence to previously known cyclotide sequences, whereas differences occurred in loop two. Analysis of the transcriptome resulted in three known and 19 new predicted cyclotide sequences. The number of sequences found in the present study is similar to previous work where transcriptome sequencing was used in cyclotide exploration^15^. Some of the masses calculated for these peptides were not found in the mass spectra of extracts from *V. anagae.* Such differences have been reported before, i.e. peptides expressed on the transcript level are not detected in the peptidome or vice versa^15,42^. Some peptides may be expressed in low abundances, or not at all, and are hence not detected by MS. Some peptides, like vian 2, can be found in high abundance on the peptide level, but not at all in the transcriptome. The explanation of these discrepancies is still unknown, but it is clear that some cyclotides are stored for longer times in the plant^43^. In the current study, a part of the explanation may also be that the sample for transcriptome analysis was not collected in the same time of the year as the rest of the samples due to technical reasons—the season (different environmental and physiological conditions) may have influenced the cyclotide expression. Nevertheless, this shows that the complete repertoire of cyclotides produced by a particular species goes beyond what can be detected at one occasion or using a single method. One solution to get the complete picture of all possible cyclotides in a given species would be whole genome sequencing. However, the function of these genes would still have to be monitored on the peptide level.

In addition to differences in cyclotide expression profiles, as judged from MS and HPLC, the cyclotide extracts from *V. anagae*, *V. cheiranthifolia* and *V. palmensis* exhibited different levels of antifungal activities. The lowest MICs against all fungal species tested was shown for the extract of *V. anagae*, which also contained the highest abundance of cyclotides in relation to other compounds. This may be associated with its cyclotide composition being similar to *V. odorata*, especially the presence of some masses corresponding to cycloviolacins that have been shown to have antifungal activity^20^. The higher antifungal potency of the *V. anagae* cyclotide extract may be interpreted as an indication of the natural target(s) of the suite of cyclotides in this particular species, as a result of its environment. It has been suggested that plant species facing a larger variety of pathogens and herbivores display a higher diversity of peptides^14^. Our results may support this hypothesis: smaller numbers of different peptides were observed in *V. cheiranthifolia* and *V. palmensis* which grow in very dry, cold conditions at high altitudes^30,35^. On the other hand, *V. odorata* and especially *V. anagae*, which reside in the Canary Islands subtropical forests^38^ display a larger variety of peptides. It would be ideal to test the antifungal activities of cyclotide extracts prepared from different populations of a species as well. Therefore, it would be possible to show, for a given species, if the environment determines not only the cyclotide composition but also the antifungal effect of the plant extract. However, most of the investigated species are either protected or the populations were small, so that collection of larger quantities of plant material, required to produce enough extract for the assays, was not possible. Therefore, in order to perform the assays, the extracts from plants belonging to different populations of a given species had to be pooled.

In the current study, we used ultrahigh resolution FTICR MALDI-MS imaging for cyclotide abundance. MALDI-MS had been successfully used to measure peptide concentration before, the method developed by Colgrave et al*.*^44^, allowed precise absolute quantification of a single cyclotide in serum. We measured the relative abundance of peptides instead of performing absolute quantification. Spectra of the whole spots of extracts were collected as an image with 200 µm spatial resolution, from which individual cyclotides were analyzed. This approach provides automation of the analysis and more data points per extract spot, which in turn results in more robust average intensities. The method is highly efficient, enabling the analysis of many samples in a short period of time (i.e. hundreds of samples in a matter of few hours) and across multiple peptides.

The current study exemplifies the applicability of cyclotides as markers in chemosystematics because different species could be distinguished on the basis of their peptide production patterns in the PCA. This analysis was built on multiple specimens from each population, each collected and analyzed individually to take into account the variability between individual plants. The analysis was qualitative and semiquantitative—different cyclotides were distinguished by their molecular mass but also by their relative quantities (signal intensities) for each peptide. The applied method allowed to select and investigate 30 different cyclotides in the mass spectra of all species and populations. The peptide pattern diversification was notable in case of the *V. cheiranthifolia* Guajara population, which was clearly distinct from the others. This result supports the genetic differentiation of this population shown by Rodríguez-Rodríguez et al*.*^30^. Recently, on the basis of morphological features, this population was proposed to be separated into a new species—*V. guaxarensis*^29^. Interestingly, all sampled individuals of the Guajara population grouped with *V. palmensis* in the PCA, not with other *V. cheiranthifolia* populations. The two species, *V. palmensis* and *V. cheiranthifolia*, are closely related to each other^30,35^. According to Rodríguez-Rodríguez et al*.*^30^, the Guajara population is the oldest and presumably other *V. cheiranthifolia* are successors. The results of the present study lend support to that hypothesis. On the contrary, *V. palmensis* showed homogeneity in terms of cyclotide patterns among studied populations. These findings may also corroborate a previous study, which showed low genetical variability between *V. palmensis* populations (Batista and Sosa^35^).

The above demonstrates the importance of both peptide identity and abundance (i.e. quantity) for chemosystematic applications. In fact, differences in abundance of only two peptides have been shown to be enough to distinguish the species *V. odorata* and *V. uliginosa*. They produce very similar set of cyclotides^15,31^ but differ in quantities of cycloviolacins cyO2 and cyO13. Both peptides are produced by both violets, but in *V. uliginosa* cyO2 is a minor peptide and cyO13 one of the major and vice versa in *V. odorata*^15,31^. The species could be distinguished on this basis regardless of the growth conditions. CyO13 was shown to be the main cycloviolacin in *V. uliginosa* even under treatment of different plant growth regulators in in vitro culture^15^. To expand this type of analysis as done in the present study (in particular in combination with the robustness and speed of the MALDI-MS assay) may prove to be a very useful tool for chemosystematic investigations.

Analyses of multiple samples from individual plants and populations also showed variations in cyclotide patterns. This raises the question: is this variability in cyclotide expression within species based on genetic variations or environmental factors? Both arguments can be made. To the best of our knowledge, no literature about the genetics, ecology or physiology of *V. anagae* is available. However, considering the differences in cyclotide production between populations seen in the current analysis, some genetical variation can be hypothesized. It seems less likely that the differences reflect the environment influences as the populations are located in close proximity. *V. anagae* appears to propagate asexually; during several visits to the sites at different times of the year (December—late May), chasmogamous flowers were observed but never seed capsules (BS pers. obs.). In this case, there would be no gene flow between populations, genetical variability could be preserved, despite proximity of the locations.

In case of *V. odorata*, the differences in cyclotide production patterns between populations, shown in the current study, may to some degree reflect the environmental influences. The samples were obtained from populations at distant locations residing from very different habitats. Little is known about the genetic variation between the *V. odorata* populations residing at the archipelago. However, some level of homogeneity can be hypothesized. In Marcussen 2006 it was shown, on the basis of selected molecular markers, that *V. odorata* from Tenerife was genetically similar to the continental populations^45^. *V. odorata* is a common species, not endemic to the Islands. It can be presumed that it arrived to the islands relatively recently, making differentiation and speciation unlikely. Therefore, it can be presumed that the genetical variability is low between populations residing at the islands. Specific cyclotides are produced and accumulated in plant organs and tissues to target specific types of pathogens or insect herbivores^20,23^. Therefore, the minor differences in suits of cyclotides produced by *V. odorata* shown in the current study may reflect the response of the plant to conditions/types of stress present in particular habitat. However, more data, e.g. from transplant experiments would be required to rule out how the genetical and environmental factors contribute to the observed inter-populations differences.

## Conclusion

The present study showed the diversity of cyclotides produced by violets from the Canary Islands. Using this as an example, it is demonstrated how the production patterns may reflect inter-species and inter-population variations and that cyclotide analysis can be utilized in chemotaxonomy. The present study supports the hypothesis that specific suits of peptides evolved for the needs of different species and their environments.

## Methods

### Plant material

**Figure 7:**
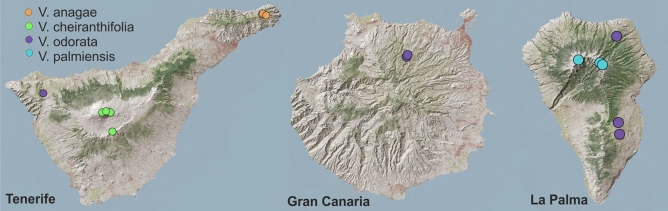
The topography of the three islands of the Canary Islands Archipelago (Tenerife, Gran Canaria, La Palma) with marked sampling locations of 4 viola species: *V. anagae*,* V. cheiranthifolia*,* V. odorata*,* V. palmensis*. The outline was created using ArcGis 10.7.1 software (https://desktop.arcgis.com/en/arcmap/) and LIDAR map of the Canary Islands (https://www.idecanarias.es/node/119).

Four *Viola* species were collected from three Canary Islands-Tenerife, Gran Canaria and La Palma. *Viola anagae* Gilli and *V. cheiranthifolia* Humb. & Bonpl*.* were sampled on Tenerife,* V. palmensis* Webb & Berth on La Palma and *V. odorata* L. on all three islands. Sixteen locations were sampled: three locations each for *V. anagae* and *V. palmensis*, four for *V. cheiranthifolia*, and six for *V. odorata.* The collection of all plant material took place in May 2019. From each location at least five individuals (above ground parts) were sampled, desiccated on silica gel and stored at room temperature. One additional population of *V. anagae* was sampled in January 2020 for transcriptome sequencing. A whole plant was collected and living sample was shipped to Uppsala (Sweden). Fresh tissues (fragments of a leaf, petiole and root) were transferred to RNAprotect Tissue Reagent (Qiagen, Düsseldorf, Germany) immediately after arrival and stored at − 20 °C. The collection sites are outlined in Fig. 7, and the details of the locations with GPS coordinates are compiled in Supplement 4. The endemic, protected species were collected from respective national parks under permissions number: O00006501e1900035563 (*V. anagae*, Servicio Administrativo de Medio Ambiente-LA0008198/Cabildo Insular de Tenerife); O00006501s1900022961 (*V. cheiranthifolia*, Parque Nacional del Teide); PTSS/5929/2019 (*V. palmensis*, Parque Nacional de La Caldera de Taburiente). As the permissions allowed sampling only leaf samples, the whole plants were not collected. The reference samples (dried leaves and original frozen extracts) for each of the species and population together with materials allowing identification of the species (photographic documentation including photos with GPS coordinates, time and date stamps, taken upon collection) are deposited at Pharmacognosy, Faculty of Pharmacy, Uppsala University and made available upon request sent to the corresponding author. All methods were performed in accordance with the relevant guidelines/regulations/legislation.

### Preparation of extracts and MALDI-MS analysis

Dried leaves from all specimens were placed in a separate 2 ml Eppendorf tubes containing a metal bead and powdered using a TissueLyser (Qiagen, Germantown, MD), 1 min at 25 Hz. Subsequently, 10 mg of each powdered sample was transferred to a separate 2 ml Eppendorf tube with a metal bead and extracted in a TissueLyser for 2.5 min at 25 Hz, with 1 ml 30% acetonitrile (ACN), 0.1% trifluoroacetic acid (TFA) in MiliQ water. Tubes were centrifuged and supernatant was collected.

To match the concentrations with linear response range of matrix-assisted laser desorption/ionization—mass spectrometry (MALDI-MS), all the samples were diluted sixfold. The diluted extracts (0.5 µl of each sample) were spotted on a metal plate and air dried. The plates were sprayed with a MALDI matrix solution (2,5-dihydroxybenzoic acid, 35 mg/ml in 50% ACN, 0.2% TFA) using an automatic matrix sprayer (TM-Sprayer; HTX Technologies, Chapel Hill, NC) at a flow rate of 70 µl/min with a spray nozzle velocity of 1100 mm/min, track spacing of 2 mm (criss-cross pattern), nitrogen pressure of 6 psi, and a spray nozzle temperature of 90 °C for 8 passes. All the spots were analyzed using a MALDI Fourier-transform ion cyclotron resonance (FTICR) (solariX 7T-2ω, Bruker Daltonics, Bremen, Germany) mass spectrometer equipped with a Smartbeam II 2 kHz laser operated in positive ionization mode. The spot areas were imaged at 250 µm lateral resolution. The relative quantitative analysis for monoisotopic cyclotide ions—the mean intensity per pixel per spot (approx. 70 pixels per spot)—was performed in msIQuant software^46^. Spectra were normalized against the root mean square (RMS) of all data points. Monoisotopic ions, not overlapping with others in the average spectra were selected. Additionally, serial dilution of extracts from different species were also spotted on a MALDI-MS target plate to assess the range of dilutions in which the relation of concentration to intensity is linear for a given ion. Ions showing a linear relation for any of the species and being in the linear range in the used extract dilutions were included in the analysis. Average mass spectra were generated in FlexImaging, version 4.0 (Bruker Daltonics). The cyclotides produced by a particular species were distinguished by their monoisotopic molecular mass in between 2.8 and 3.8 kDa.

Principal component analysis (PCA) was performed for the data collected with MALDI-MS. The intensities of selected monoisotopic + 1 ions corresponding to cyclotides were used for the PCA and displayed according to differences between species or locations. For each cyclotide, a Kruskal–Wallis rank sum test followed by a Nemenyi post-hoc test was performed to test for differences in cyclotide variance in the different species^47^. All statistical analyses were conducted in R. Significance testing was done using the package PMCMRplus and plots were produced with the package ggplot2^48–50^. UPGMA clustering analysis of the cyclotides based on Euclidean distances was visualised with a heatmap prepared in R (https://CRAN.R-project.org/package=pheatmap)^51^.

### *V. anagae* transcriptome sequencing and cyclotide mining

RNA extraction and transcriptome sequencing were performed by Macrogen Inc. (Seoul, South Korea). The sample was shipped on ice, preserved in RNAprotect Tissue Reagent (Qiagen). Libraries were prepared using TruSeq stranded mRNA kit (Illumina, San Diego, USA), followed by Illumina Novaseq 2 × 100 bp paired-end sequencing. The transcriptome was de novo assembled using Trinity^52^. The assembled transcriptome was mined for similar sequences to the cyclotides from Cybase (retrieved 03.03.2019; http://www.cybase.org.au/) using the standalone NCBI-BLAST + service in the Ugene software package (v.1.31.0). The results of BLAST search were combined with records resulting from a motif search (C-x(0,1)-[ES]-S-C-[AV]-[MFYW]-I-[PS]-x(0,1)-C) performed using Fuzzpro of EMBOSS (v. 5.0.0)^53,54^. Duplicates were removed from the combined database and extracted open reading frames were aligned using Clustal Ω^55^. A sequence was considered a cyclotide if the six conserved cysteines aligned with previously known cyclotides and if it contained aspartic acid (D) or asparagine (N) at the C-terminal.

### LC-MSMS sequencing

A sample of 650 mg of *V. anagae* ground plant material was prepared by extracting twice with 10 ml of 30% ACN with 0.05% TFA and once with 10 ml of 60% ACN in 0.05% TFA. The extract was first purified on an ÄKTA basic HPLC system (Amersham Biosciences, Uppsala, Sweden) with a SNAP Ultra C_18_ flash column, 20 µm, 100 Å, 12 g (Biotage) on a 20 min gradient from 10 to 70% ACN in 0.05% TFA with a flowrate of 10 ml/min. Fractions were analyzed by electrospray (ESI) MS in positive ionization mode (Finnigan LCQ ion trap, Thermo Electron Co., Waltham, MA, USA). The cyclotide containing fractions were lyophilized and subsequently re-dissolved in 10% ACN (0.05% TFA). Cyclotides were isolated using RP-HPLC using a Shimadzu LC20 system (Shimadzu Scientific Instruments, Kyoto, Japan) equipped with a Jupiter 5 µm C_18_ 300 Å, 250 × 10 mm column (Phenomenex). Reduction, alkylation and cleavage of the peptides to be sequenced was performed as described before^15,56^ and the primary structure of the peptide fragments was analyzed using UPLC-QToF nanospray MS (Waters nanoAcquity, QToF Micro; 75 µm × 250 mm 1.7 µm BEH130 C18). MS scan window was set to 200–2500 m*/z* and for MS/MS to 50–2000 m*/z*.

### Antifungal assays

Cyclotide extracts of each *Viola* species were prepared by grinding 350–450 mg of dry plant material. Subsequently, the material was extracted twice with 10 ml of 30% ACN (0.05% TFA) in water and once with 10 ml of 60% ACN in 0.05% TFA. The extracts were combined, lyophilized, and resuspended in 2.5 ml Milli-Q water, and desalted using size exclusion chromatography (PD-10, GE Healthcare). The cyclotide extract composition was assessed by UV-RP-HPLC using a Shimadzu LC20 system described above and a 40 min gradient from 5 to 55% of ACN (0.05% TFA). The resulting extracts were then lyophilized and weighted.

The enriched cyclotide extracts from *V. anagae*, *V. palmensis* and *V. cheiranthifolia* were tested against five fungal plant pathogens: *Aspergillus fumigatus* Fresenius, *Aspergillus pseudonomius* Varga, *Mycosphaerella fragariae* (Tulasne) Lindau, *Botrytis cinerea* Pers.; Fr. and *Fusarium culmorum* Sm. Sacc. *B. cinerea*, *F. culmorum* and *M. fragariae* were received from the Institute of Plant Protection—National Research Institute, Poznań, Poland (accession no. 2235, 2169, 1089 respectively), *A. pseudonomius* from Westerdijk institute, Netherlands (CBS 119388) and *A. fumigatus* strain J7, as previously described^57^. Stock suspensions of the fungal strains were prepared using established procedures^20,57^. All cultures were maintained at room temperature (RT) and under aseptic conditions.

Standard microdilution assays in flat-bottomed 96-well Nunc A/S plates (Thermo Scientific) were performed^58,59^. The assays were performed in 24 g l^−1^ potato dextrose broth (PDB, Sigma-Aldrich St. Louis, MO, United States) for *B. cinerea*, *F. culmorum* and *M. fragariae*; and RPMI 1640 medium for both *Aspergillus* species. The cyclotide enriched plant extracts were dissolved in the respective media at starting concentrations between 0.4 and 1 mg l^−1^. The minimal inhibitory concentration (MIC) was assessed by measuring the optical density (OD, absorbance at 600 nm) at the timepoint of inoculation (0 h) and again after 48 h (for *M. fragariae*,* B. cinerea* and *F. culmorum*) or 72 h (for *A. fumigatus* and *A. pseudonomius*), using a Varioskan Flash Multimode Reader (Thermo Scientific). The OD_48h–0h_ values lower than 0.1 indicated growth inhibition at this concentration. All antifungal assays were performed in three replicates.

## Supplementary Information

Supplementary Information 1.

Supplementary Information 2.

Supplementary Information 3.

Supplementary Information 4.
