# Supplementary material for: Cyclotide host-defense tailored for species and environments in violets from the Canary Islands
Source: Sci Rep. 2021 Jun 14;11:12452. doi: 10.1038/s41598-021-91555-y (PMC8203695; doi:10.1038/s41598-021-91555-y)
Supplement: Supplementary file 3 — Supplementary Information 3. [file 41598_2021_91555_MOESM3_ESM.docx]

**Title:**

Cyclotide host-defense tailored for species and environments in violets from the Canary Islands

**Authors:** Blazej Slazak*^1, 2^, Klara Kaltenböck*^2, 3^, Karin Steffen^2^, Martyna Rogala^4^, Priscila Rodríguez^5^, Anna Nilsson^6,7^, Reza Shariatgorji^6,7^, Per E. Andrén^6,7^, Ulf Göransson^2^

Supplement 3. Additional analysis of the average relative abundances of 30 selected cyclotides, across all the investigated violet species: *V. anagae*, *V. cheiranthifolia*, *V. odorata*, *V. palmensis*

(**A**) PCA analysis of the dataset for all investigated species, subset of species and among populations (locations) of a particular species – PC1 and PC3 shown. Single point indicates individual sampled plant.

(**B**) Symmetric heatmap presentation of the data based on Euclidean distances and UPGMA clustering prepared using the pheatmap package in R (<https://CRAN.R-project.org/package=pheatmap>) ^1^. The darker the color, the more similar two samples are in their overall cyclotide production pattern.
