# Supplementary material for: Cyclotide host-defense tailored for species and environments in violets from the Canary Islands
Source: Sci Rep. 2021 Jun 14;11:12452. doi: 10.1038/s41598-021-91555-y (PMC8203695; doi:10.1038/s41598-021-91555-y)
Supplement: Supplementary file 4 — Supplementary Information 4. [file 41598_2021_91555_MOESM4_ESM.docx]

**Title:**

Cyclotide host-defense tailored for species and environments in violets from the Canary Islands

**Authors:** Blazej Slazak*^1, 2^, Klara Kaltenböck*^2, 3^, Karin Steffen^2^, Martyna Rogala^4^, Priscila Rodríguez^5^, Anna Nilsson^6,7^, Reza Shariatgorji^6,7^, Per E. Andrén^6,7^, Ulf Göransson^2^

Supplement 4. GPS coordinates of all sampling sites with altitude and the corresponding island.

| Species | ID | Locality | Island | Altitude  [mamsl] | Latitude  [°N] | Longitude  [°W] |
| --- | --- | --- | --- | --- | --- | --- |
| *V. anagae* | A1-6 | Site 1 | Tenerife | 886 | 28.558614 | -16.175222 |
|  | N1-5 | Site 2 |  | 864 | 28.557778 | -16.175472 |
|  | L1-5 | Site 3 |  | 876 | 28.5599827 | -16.1734920 |
|  | Transcriptome | Site 4 |  | 709 | 28.5545076 | -16.1636084 |
| *V. cheiranthifolia* | H1-6 | Montana Blanca |  | 2719 | 28.271139 | -16.617278 |
|  | O1-7 | Guajara |  | 2676 | 28.215333 | -16.611444 |
|  | D1-2 | Rambleta |  | 3627 | 28.271417 | -16.641306 |
|  | B1-5 | Refugio |  | 3285 | 28.274472 | -16.630056 |
| *V. palmensis* | E1-5 | Site_4 | La Palma | 2259 | 28.7494604 | -17.8436017 |
|  | F1-5 | Site_5 |  | 2287 | 28.7449805 | -17.8382564 |
|  | J1-5 | Site_6 |  | 2330 | 28.7551428 | -17.8884502 |
| *V. odorata* | I1-7 | La Laguna | Gran Canaria | 860 | 28.0671889 | -15.5640222 |
|  | K1-7 | Barranquillo del Tabuco |  | 822 | 28.0715250 | -15.5617389 |
|  | P1-7 | Hoya de Amalio | La Palma | 1009 | 28.599778 | -17.802556 |
|  | G1-6 | La Tablada |  | 854 | 28.624139 | -17.805111 |
|  | M1-7 | Fuente Grande |  | 774 | 28.804611 | -17.808833 |
|  | C1-5 | Pista Monte del Agua | Tenerife | 1030 | 28.327056 | -16.812556 |
